# Parturient Apoplexy

**Published:** 1892-01

**Authors:** T. D. Hinebauch

**Affiliations:** Fargo, N. D.


### PARTURIENT APOPLEXY.

By T. D. Hinebauch, M. S., V. S..

The disease known under the above name is one which usually occurs in from a few hours to three or four days (and in exceptional cases later) after calving.

The pathology of the disease is, perhaps, as little known as that of almost any disease which occurs as frequently. Many of the theories which have been advanced we believe to be entirely without foundation, or else the disease which passes under the above title will have to be divided. If the term "apoplexy" be limited in its application, it means an arrest of sense and motion due to congestion or rupture of some of the vessels of the brain. We find, however, in this disease, that motion does exist, especially in the first stages ; the senses are also retained in many instances up to a few hours preceding death. Again, if there is a rupture of some of the vessels, recovery cannot take place in so short a time as it is known to. If due to congestion, many cases would necessarily terminate in inflammation of the brain or spinal cord. This would especially be true where animals are affected for several days, say from three to six. Post-mortems do not reveal such instances. At least we have never witnessed them, nor do we remember of having ever seen any on record.

In the investigation of this disease we must look elsewhere for the results. The theory which we advance is based upon clinical and therapeutical observations on a goodly number of cases. From these observations we have endeavored to arrive at the true condition which exists. We have held six post-mortems on the brain and spinal cord, but in all of them we have failed to find any clot present, even an indication of one. Congestion of these organs was only in one case, and that had been allowed to pound its head, which it did most violently. Undoubtely a great many cases which show slight congestion on post-mortem can be traced to the constant pounding of the head, which takes place before complete coma sets in. We also believe that if post-mortems were as frequently held on the brain and spinal cord of animals that have suffered from diseases where there is almost a complete absence of peristaltic action, as there is in this disease, a similar condition of the nerve centres would be noticed.

Blood clots and violent congestion undoubtedly do exist, but it is the exception rather than the rule, and ought to classed as another disease.

The disease always appears, without exception, in deep milkers. It is an indisputable fact that the deeper the milker the more highly is the nervous system developed, and that it is proportionately more susceptible to great changes.

Previous to the act of parturition there is a storing up of nerve force to be expended during calving. Ordinarily this is done, but if the act be very easily performed (and it always is preceding parturient apoplexy) the nerve forced is not expended, and as a result, a reaction takes place, a collapse, as it were, of the nervous system. This is especially true of the periphery of the nerves. In consequence, we find that the functions of the various glands, including the mammary, cease. Peristalsis decreases very rapidly, and is entirely absent, in most cases, within a short time after the attack. The few intestinal murmurs being due to the gradual but slow formation of gas.

That digestion ceases is evident upon post-mortem. We have found medicines that were administered by the mouth several hours previous to death lying in the stomach as they arrived there.

In further support of this theory we wish to cite the fact that recovery may and does take place in an incredibly short time, and is not gradual for a day or more only in exceptional instances of the disease, where due to a clot or congestion this could not result.

From a therapeutical point of view there is also evidence which tends to the above theory. If pilocarpine be administered hypodermically in from 8 to 15 grain doses. it is noticed that a reaction soon takes place in most cases. Peristaltic action soon manifests itself, and is considerably hastened by the addition to the pilocarpine of ½ to 1 grain of salicylate of physostigmine. In my experience with these drugs in this disease, if reaction has not shortly followed their administration, death has invariably been the result. If our theory be correct, we would expect the above results from pilocarpine. Pilocarpine stimulates the periphery of efferent nerves going to glands, and hence increases their reaction. This is true of the salivary glands and the mucous glands of the respiratory and digestive tracts, the kidneys and mammary glands. It shows and dimishes blood pressure and lessens the irritability of motor nerves.

In case the disease be due to congestion, we can readily see that the pilocarpine would be beneficial from its power to relieve blood pressure.

There may be other remedies which are equally as effective in overcoming the disease as the one we have suggested, but we have never been able to apply them with as good results. We also firmly believe that any remedy by way of the mouth is simply wasted from the fact that the stomach cannot assimilate it. Many of the cases of recovery, that take place with such treatment, would undoubtedly recover if allowed to take their own course. Indeed cases have come to our knowledge where animals suffering from this disease have recovered without any treatment whatever.

FARGO, N. D.

